# A nod to paratuberculosis: NOD1 and NOD2 expression in the pathological spectrum of *Mycobacterium avium* subsp. *paratuberculosis* infection in cattle

**DOI:** 10.3389/fvets.2025.1549056

**Published:** 2025-05-13

**Authors:** David Zapico, José Espinosa, Pedro Mendívil, Miguel Criado, Julio Benavides, Miguel Fernández

**Affiliations:** ^1^Departamento de Sanidad Animal, Facultad de Veterinaria, Universidad de León, León, Spain; ^2^Departamento de Sanidad Animal, Instituto de Ganadería de Montaña (IGM), CSIC-ULE, León, Spain

**Keywords:** cattle, granuloma, lesion, paratuberculosis, NOD-like receptor, NOD1, NOD2

## Abstract

*Mycobacterium avium* subsp. *paratuberculosis* causes various types of granulomatous lesions in cattle, ranging from focal lesions associated with latency to diffuse lesions observed in animals with clinical disease. While the exact determining factors are unknown, recent evidence highlights the key role of innate immunity in the outcome of the infection. NOD-like receptors, which are innate immune proteins, play a significant role in recognizing intracellular pathogens, including mycobacteria. This study aimed to evaluate the expression of NOD1 and NOD2 in intestinal samples from cattle with different types of lesions associated with paratuberculosis: focal, diffuse paucibacillary, and multibacillary forms. The expression of NOD1 and NOD2 was assessed according to the number of immunolabeled cells, and only those cells consistent with macrophages were considered. A significant increase in the number of NOD1+ and NOD2+ macrophages was observed in cattle with diffuse multibacillary forms compared to the other groups. No expression of NOD1 or NOD2 was detected in the focal and diffuse paucibacillary lesions, while a strong expression of NOD2 and occasional NOD1 was observed in the multibacillary granulomas. These findings suggest that NOD1 and NOD2 are involved in the pathogenesis of bovine paratuberculosis.

## 1 Introduction

Paratuberculosis is a chronic inflammation of the small intestine of cattle and other ruminants, caused by *Mycobacterium avium* subsp. *paratuberculosis* (MAP) (1). The disease causes important economic losses in the livestock industry, associated with lower production and premature culling of infected animals (2, 3). Calves get exposed to MAP at a young age via the fecal–oral route (4), which can result in a range of outcomes, such as complete clearance of the pathogen, lifespan asymptomatic infection, or clinical disease in adulthood (5). The infected cattle may show different types of intestinal lesions associated with the infection, from focal granulomas in Peyer’s patches to diffuse granulomatous enteritis (6), which shares a relation with the stages of the disease (7). Many aspects of its pathogenesis are still poorly understood, in particular, the factors that determine the host response to infection. Nonetheless, there is recent evidence suggesting a central role of innate immunity in the susceptibility/resistance of cattle to paratuberculosis (8, 9). Several lymphocyte and macrophage subpopulations have been evaluated in relation to MAP-associated lesions in cattle (10–16), but the markers of innate immunity have been less explored (17, 18).

Pattern recognition receptors (PRRs) play a critical role in the detection of pathogens by innate immune cells and in the onset of specific responses (19, 20). Among these, nucleotide-binding oligomerization domain (NOD)-like receptors (NLRs) have emerged as key components of the immunological response against intracellular bacteria (21). NOD1 and NOD2 are cytosolic NLRs that, respectively, sense meso-diaminopimelic acid (m-DAP)-containing peptides and muramyl dipeptide (MDP) (22, 23), which constitute motifs of the mycobacterial peptidoglycan (PGN) (24). These receptors are expressed by a wide variety of cells in the intestine (25, 26) and were reported to detect MAP ligands (27). NOD1 participates in the recognition of MAP by intestinal epithelial cells (28), but its role in the innate response of bovine macrophages is unclear (29). NOD2 gene polymorphisms have been associated with susceptibility to paratuberculosis infection in cattle (30–32). Furthermore, variations in the patterns of NOD2 expression were detected in the intestine of infected sheep with different pathological forms (33). Nevertheless, the precise role of NOD1 and NOD2 in the pathogenesis of paratuberculosis remains unclear.

The aim of this study was to evaluate the expression of NOD1 and NOD2 in the different types of intestinal lesions associated with bovine paratuberculosis, using immunohistochemical techniques.

## 2 Materials and methods

### 2.1 Samples

Paraffin-embedded formalin-fixed (10% neutral buffered formalin) tissue samples from the intestine (jejunum, which includes Peyer’s patches, and ileum) and mesenteric lymph nodes of 20 female Holstein cattle, aged 1–6 years, were selected for histopathological analysis. These animals were from two commercial dairy herds where a follow-up study on losses due to paratuberculosis was ongoing. The cattle were culled in an authorized slaughterhouse for productive reasons, in compliance with current legislation. Paratuberculosis infection was confirmed by both bacteriological culture of frozen tissue samples and nested-PCR to detect MAP DNA as previously described (6). Samples that tested negative for both methods were used as negative controls for the study.

Tissue sections, 2.5 μm in thickness, were obtained and stained using Harris’s hematoxylin and eosin (H&E) for general histological examination, and the Ziehl–Neelsen method was used to identify acid-fast bacilli (AFB). Histological examination revealed no lesions consistent with MAP infection in the five cattle that tested negative by both bacteriological culture and nested PCR. In contrast, granulomatous lesions were identified in 15 animals that tested positive for MAP infection.

According to the lesions, animals were histologically classified as *focal* (*n* = 5), *diffuse paucibacillary* (*n* = 5), and *diffuse multibacillary* (*n* = 5) (Supplementary Figure S1), following the guidelines provided by Gonzalez et al. (6). *Focal lesions* consisted of well-demarcated granulomas, formed by 5–30 epithelioid macrophages, at the interfollicular zone of the Peyer’s patches or lymph nodes. *Diffuse lesions* were characterized by diffuse lymphadenitis and enteritis that varied in the type of inflammatory infiltrate and the amount of AFB. *The diffuse paucibacillary type* consisted of a diffuse lymphocytic infiltrate, with some well-defined granulomas among the lymphocytes. AFB was either undetected or present in minimal amounts. *Diffuse multibacillary type* consisted of a severe granulomatous infiltrate, composed of macrophages harboring large numbers of AFB.

### 2.2 Immunohistochemistry

A total of 20 tissue sections of the intestine and 20 sections of the regional lymph nodes, one from each animal included in the study, were selected and immunolabeled using rabbit IgG isotype anti-NOD1 (PA5-17328, Invitrogen™, Waltham, Massachusetts, USA) and anti-NOD2 (BS-7084R, Bioss Inc., Woburn, Massachusetts, USA) polyclonal primary antibodies at 1:200 and 1:300 dilution titers, respectively. Heat-mediated antigen retrieval was achieved using the PT Link system (Dako-Agilent® technologies, Santa Clara, USA) for 20 min at 95°C with target retrieval solution pH 9 for NOD1 and pH 6 for NOD2. The immunohistochemical technique was performed as described elsewhere (10). Appropriate species- and isotype-matched immunoglobulins were used as negative controls.

The specificity of the NOD1 primary antibody was tested by Western blot analysis of cattle buffy coat cells stimulated *in vitro* with concanavalin A as previously described (17), as no proven or predicted reactivity in the bovine species is reported in the manufacturer’s instructions. Blocking was performed with Tris-phosphate buffer with 0.05% Tween-20 (TBS-T) containing either 5% non-fat milk or 5% bovine serum albumin (BSA). NOD1 primary antibody diluted in TBS-T containing 0.5% non-fat milk or 3% BSA (according to the blocking buffer) and appropriate goat anti-rabbit (YH381824, Invitrogen, Waltham, MA, USA) horseradish peroxidase-conjugated secondary antibody diluted in TBS-T were used at 1:500. In the case of the NOD2 antibody, no additional validation was performed, as the datasheet predicts a cross-reactivity in cattle, based on the high degree of homology (81.2%) between human and bovine NOD2 proteins (34).

### 2.3 Evaluation of the immunolabeling

Due to the heterogeneous nature and distribution of the immunolabeled cells, a differential cell count was performed on the lamina propria (LP), gut-associated lymphoid tissue (GALT), and mesenteric lymph node (LN) of each section evaluated in the study. In each slide, 30 randomly chosen fields were selected from each of the two intestinal layers analyzed and the lymph node and photographed at 400x magnification (Nikon® Eclipse E600 microscope with a Nikon® DS-Fi1 digital camera). The type of cell stained was assessed according to morphological features, and only those with clear macrophage morphology were considered. In addition, the distribution of the positively immunolabeled cells in relation to MAP-associated granulomatous lesions was also assessed.

Blinded evaluation of the immunostaining was assessed independently by two pathologists (D.Z. and M.F.), and discordant results were discussed in a multi-headed microscope to reach consensus.

### 2.4 Statistical analysis

Cell counts for NOD1 and NOD2 were expressed as means, standard deviation, and range (minimum and maximum) following conventional statistical descriptive procedures. The Kolmogorov–Smirnov test was used to evaluate the normality of the data. Since the values obtained did not fit a normal distribution and could not be statistically transformed, non-parametric tests were used. To compare the number of immunolabeled cells between the different infection statuses (control, infected), lesion types (control, focal, diffuse paucibacillary, and diffuse multibacillary), and intestinal locations (LP, GALT, and LN), the non-parametric Mann–Whitney U-test and Kruskal–Wallis tests were used. In a second step, to assess which pair of groups had the differences present, a *post-hoc* analysis (pairwise Wilcoxon rank-sum) was performed with Bonferroni correction for the level of significance (35). *p*-values of < 0.05 were considered statistically significant.

All statistical analyses were performed with the R software version 3.5.3 (R Foundation, Vienna, Austria).

## 3 Results

### 3.1 Distribution of the immunolabeled cells

The positively immunolabeled cells had markedly brown-colored cytoplasm and their identification was made according to morphological features. The immunolabeled cells for NOD1 and NOD2 in the stroma had moderate ameboid-shaped cytoplasm and were morphologically consistent with macrophages, although the presence of NOD1+ and NOD2+ cells with small cytoplasm and multilobulated nuclei, compatible with neutrophils, was also detected. Triangular-shaped epithelial cells, consistent with Paneth cells, showed positive immunoreactivity for NOD1 and NOD2 in the crypts of Lieberkühn.

**Figure 1:**
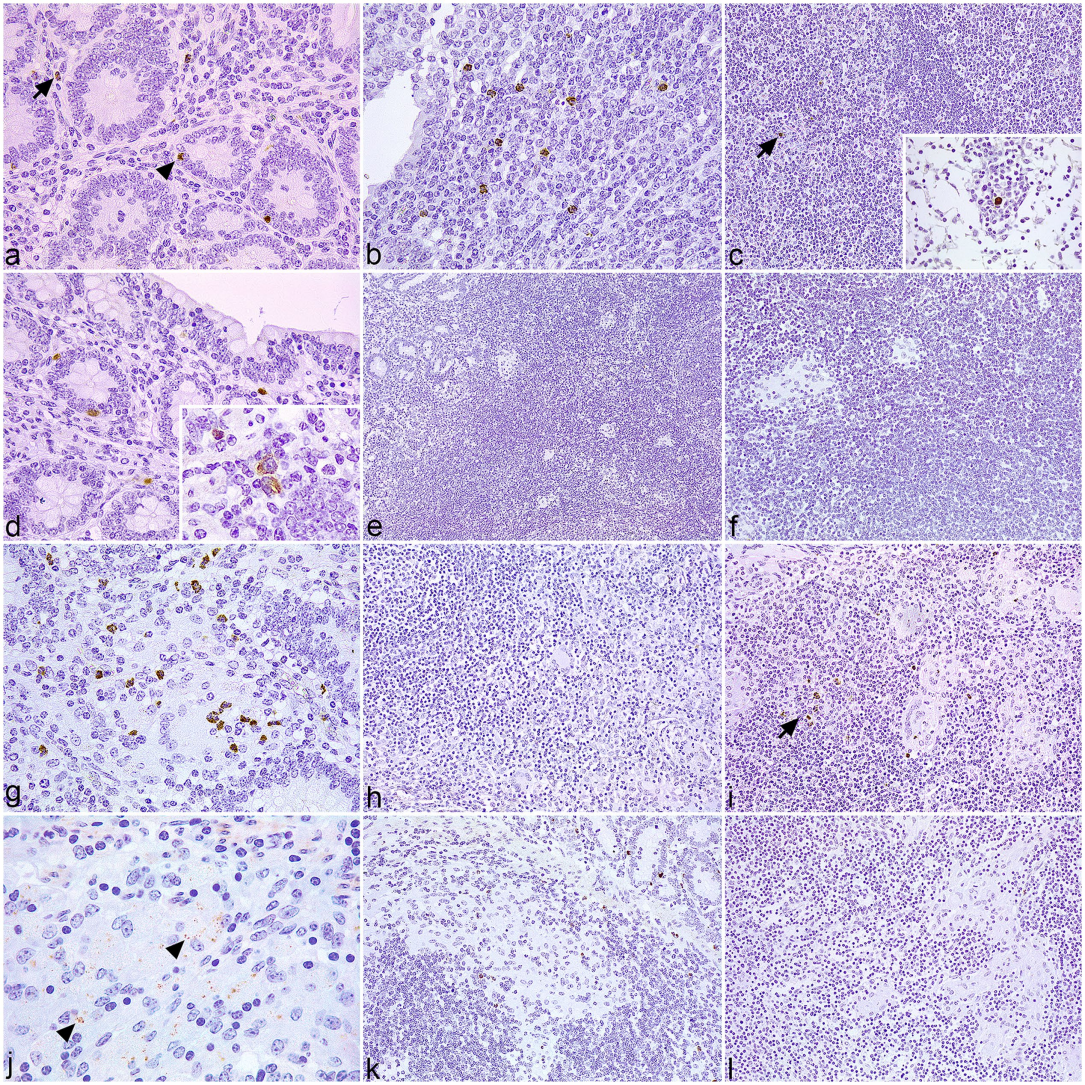
Tissue sections of control and infected cattle showing different types of lesions associated with paratuberculosis infection, immunolabeled for NOD1. **(a–c)** Sections of the jejunum and jejunal lymph node (LN) of uninfected control cattle. **(a)** Few immunolabeled macrophages for NOD1 (arrow) are present in the stroma of the lamina propria (LP). Paneth cells show positive immunoreactivity in the crypts (arrowhead). **(b)** Group of NOD1 + macrophages situated in the dome of a Peyer’s patch. **(c)** Scant positive cells for NOD1 (arrow) in the cortex of a mesenteric LN. Insert: Labeled macrophage in a medullary cord. **(d–f)** Sections of the jejunum and jejunal LN of cattle with focal lesions. **(d)** Few macrophages and Paneth cells display NOD1 + staining in the LP. Insert: Detail of NOD1 + cytoplasmic staining in macrophages. **(e)** Absence of NOD1 expression in the granulomas present in Peyer’s patches. **(f)** Lack of NOD1 + macrophages in the focal lesions situated in the LN. **(g–i)** Sections of the ileum and jejunal LN of cattle with diffuse paucibacillary lesions. **(g)** Moderate numbers of immunolabeled neutrophils are in close relationship to a granuloma present in the LP, which does not stain for NOD1. **(h)** The epithelioid and Langhans giant cells infiltrating the gut-associated lymphoid tissue show no immunoreactivity for NOD1. **(i)** Absence of NOD1 + staining in the granulomatous lesions situated in the LN. Few positive PMNs are present (arrow). **(j–l)** Sections of the ileum and jejunal LN of cattle with diffuse multibacillary lesions. **(j)** Scarce punctate peroxidase reaction for NOD1 (arrowheads) in the cytoplasm of the epithelioid cells invading the LP. **(k)** Lack of NOD1 expression by the macrophages infiltrating the Peyer’s patches, despite a few positively immunolabeled PMNs. **(l)** The granulomatous lesions present in the LN show no immunoreactivity for NOD1.

**Figure 2:**
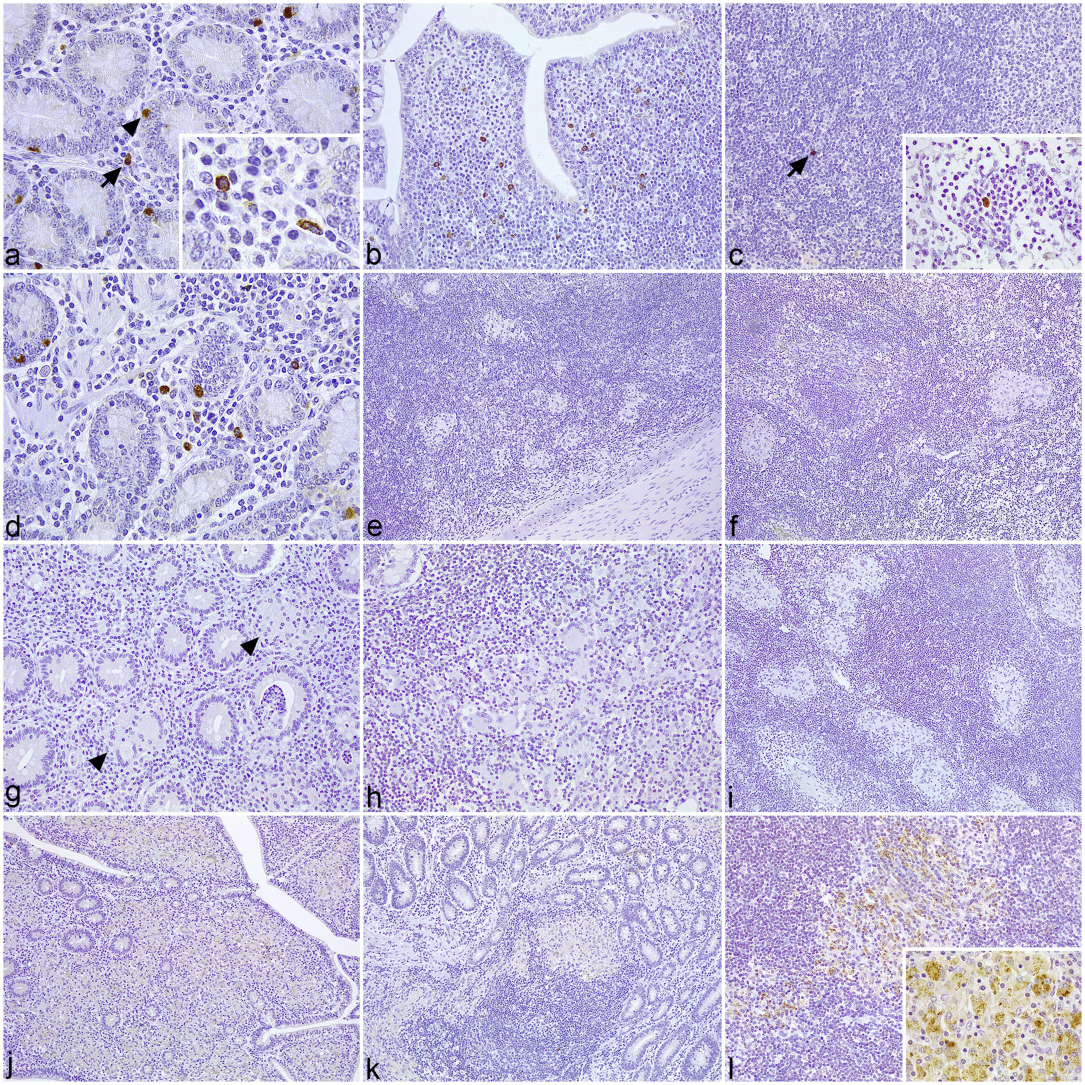
Tissue sections of control and infected cattle showing different types of lesions associated with paratuberculosis infection, immunolabeled for NOD2. **(a–c)** Sections of the jejunum and jejunal lymph nodes (LN) of uninfected control cattle. **(a)** Positively immunolabeled macrophages for NOD2 (arrow) are scattered at the lamina propria (LP) between the crypts, which show positive immunoreactivity for NOD2 in the Paneth cells (arrowhead). Insert: Detail of NOD2+ macrophages. **(b)** Several macrophages labeled for NOD2 concentrate in the dome of a Peyer’s patch. **(c)** Isolated NOD2+ macrophage (arrow) in the cortex of mesenteric LN. Insert: Solitary macrophage labeled for NOD2 in a medullary cord. **(d–f)** Sections of the jejunum and jejunal LN of cattle with focal lesions. **(d)** Positive immunoreactivity for NOD2 in the macrophages and Paneth cells located in the LP. **(e)** Lack of NOD2+ immunostaining in the granulomas situated in the Peyer’s patches. **(f)** The macrophages forming the focal lesions in the LN are not immunolabeled for NOD2. **(g–i)**. Sections of the ileum and jejunal LN of cattle with diffuse paucibacillary lesions. **(g)** Lack of immunoreactivity for NOD2 antibody in the granulomatous lesions (arrowheads) present between the lymphocytic inflammation at the LP. **(h)** The epithelioid and Langhans giant cells infiltrating the gut-associated lymphoid tissue show no immunoperoxidase reaction for NOD2. **(i)** Absence of NOD2+ immunolabeling in the granulomatous infiltrate invading the cortex of a LN. **(j–l)** Sections of the ileum and jejunal LN of cattle with diffuse multibacillary lesions. **(j)** Positive immunoreactivity for NOD2 in the granulomatous infiltrate invading the LP. **(k)** Macrophages displaying mild NOD2+ immunolabeling in the infiltrate affecting the Peyer’s patches. **(l)** Moderate to intense expression of NOD2 in a granulomatous lesions located in the cortex of a LN. Insert: Detail of the punctate to granular staining in the cytoplasm of the epithelioid cells.

In samples of control cattle, scattered macrophages, neutrophils, and individual Paneth cells showed granular cytoplasmic staining for NOD1 (Figure 1a) and NOD2 (Figure 2a) at the intestinal LP. In the associated Peyer’s patches, macrophages immunolabeled for NOD1 (Figure 1b) and NOD2 (Figure 2b) were concentrated at the dome, below the M-cell epithelium, with scarce positive cells in the interfollicular region. Fewer isolated NOD1+ (Figure 1c) and NOD2+ (Figure 2c) macrophages were present in the cortex, medullary cords, and sinus of the regional LN. In samples with focal lesions, the immunolabeled cells for NOD1 (Figure 1d) and NOD2 (Figure 2d) followed a similar distribution to the controls outside of the granulomas. The epithelioid macrophages forming the focal lesions in Peyer’s patches and mesenteric LN showed no immunoreactivity for NOD1 (Figures 1e,f) or NOD2 (Figures 2e,f). In sections with diffuse paucibacillary forms, the epithelioid and Langhans giant cells forming the granulomatous lesions among the lymphocytic infiltrate neither stain for NOD1 (Figures 1g–i) nor for NOD2 (Figures 2g–i). However, in samples with diffuse multibacillary lesions, the epithelioid cells invading the LP, GALT, and LN showed diffuse granular staining for NOD2 in the cytoplasm (Figures 2j–l), with occasional punctate immunolabeling for NOD1 (Figure 1j). In tissue sections with diffuse forms, both paucibacillary and multibacillary, variable numbers of NOD1 + neutrophils were seen in close relationship to the granulomatous lesions present in the LP, Peyer’s patches, and associated LN (Figures 1g,i,k).

### 3.2 Number of immunolabeled cells

**Figure 3:**
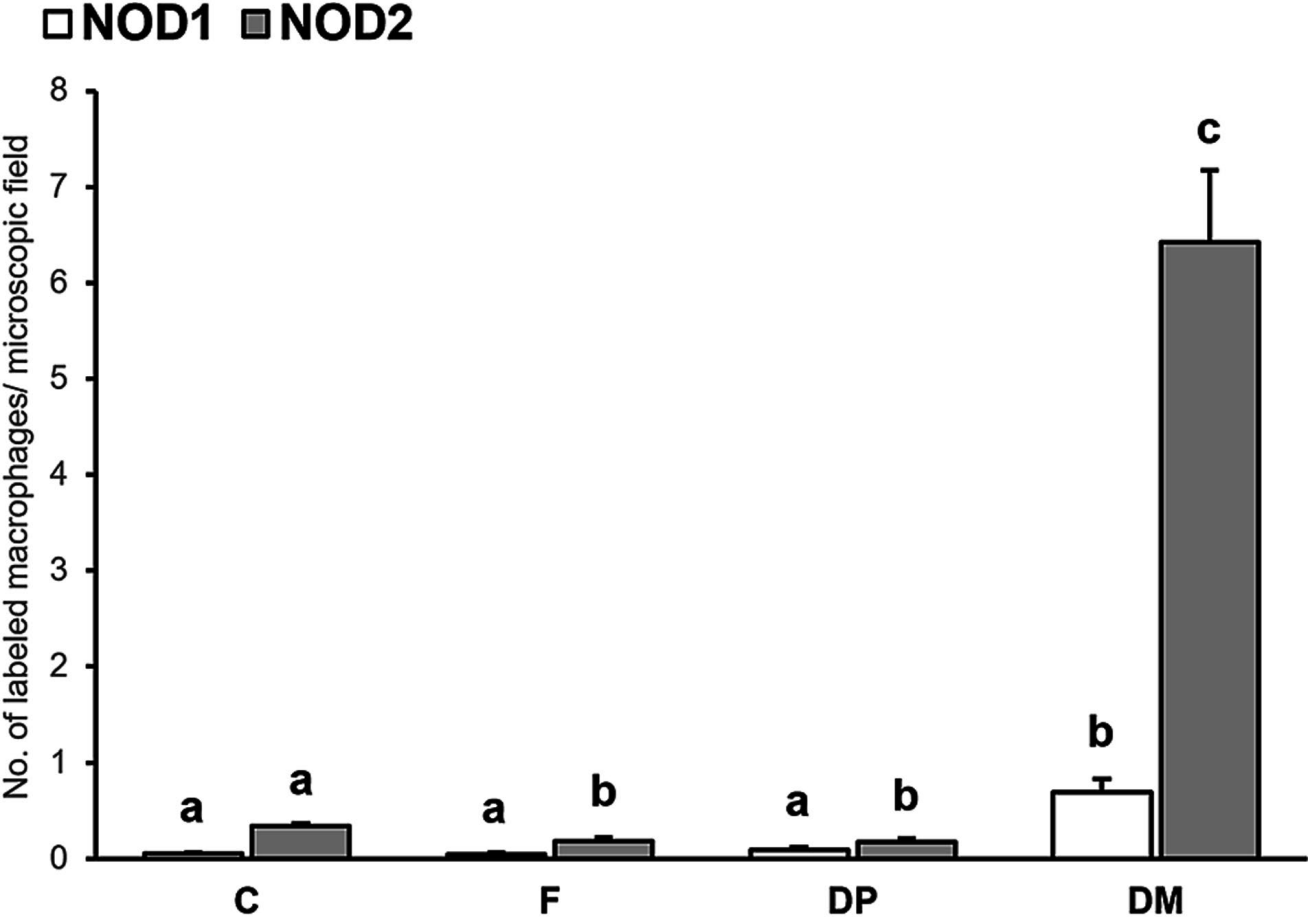
Mean number of immunolabeled macrophages for NOD1 and NOD2 in the intestine according to the type of lesion (C, control; F, focal; DP, diffuse paucibacillary; DM, diffuse multibacillary). Superscript letters indicate statistical significance. Error bars: standard error.

Only those immunolabeled cells for NOD1 and NOD2 that showed clear macrophage morphology were considered for the cell counting. A significant increase in the number of positively immunolabeled macrophages for NOD1 (*p* < 0.01) and NOD2 (*p* < 0.05) was detected in infected cattle compared to the controls. When analyzing the different lesion categories (Figure 3), the highest number of macrophages immunolabeled for NOD1 and NOD2 was observed in cows with diffuse multibacillary forms, showing significant differences compared to the rest of the groups (*p* < 0.001). Control cattle had significantly more NOD2 + macrophages compared to those with focal and diffuse paucibacillary forms (*p* < 0.001), but these two groups showed no difference (*p* > 1.000).

Regarding the different intestinal locations analyzed (LP, GALT, LN), a heterogeneous distribution of immunolabeled cells for NOD2, but not for NOD1, was observed in the three compartments. Control cattle showed a higher number of NOD2 + macrophages in the GALT compared to the LN (*p* < 0.001) and the LP (*p* < 0.05). In the group with focal lesions, a significant increase in macrophages immunolabeled for NOD2 was detected in the LP compared to the GALT (*p* < 0.01). In cattle with diffuse lesions, both paucibacillary and multibacillary, the number of NOD2 + macrophages was higher in the LP and the GALT compared to the LN (*p* < 0.001).

### 3.3 Immunoblot analysis

The specificity of the NOD1 primary antibody used for immunohistochemistry was tested by immunoblotting. Validation was confirmed by the presence of a specific band of approximately 100 kDa, consistent with the detection of NOD1 protein (107 kDa) (34, 36), without any cross-reacting bands (Supplementary Figure S2).

## 4 Discussion

Paratuberculosis leads to the development of different types of granulomatous lesions in the intestine, depending on the outcome of the infection, from focal lesions associated with early infection or latency to diffuse forms related to clinical disease (6, 37). These pathological forms show a progressive and dynamic nature (6, 39), which can be influenced by different factors such as the dose and time of the initial exposure, age, sex, genetics, and other factors affecting host immunocompetence (e.g., gestation, parturition, and negative energy balance) (40). Recent evidence has highlighted the vital role of innate immunity in the outcome of MAP infection (8, 9), although the majority of these investigations constitute candidate gene association studies or *in vitro* assays of infection. NOD1 and NOD2 belong to the NOD-like receptor family, a group of innate immune proteins involved in the recognition of intracellular pathogens (21, 38). These receptors have previously been associated with bovine paratuberculosis (29–32), but their role in the pathogenesis of the disease remains to be elucidated. The immunohistochemical analysis of NOD1 and NOD2 expression in cattle with different types of lesions associated with paratuberculosis provides a snapshot of the immunological events ongoing in the intestine of these animals at the moment of euthanasia, allowing the characterization of the immune cell populations expressing these receptors and their distribution in relation to MAP-associated granulomas.

Diffuse multibacillary forms are the most common type of lesion reported in adult cattle with clinical signs of Johne’s disease (6). These pathological forms are characterized by an anti-inflammatory micro-environment within the granulomas that allows the unrestricted growth of MAP in the macrophages (12, 14). The epithelioid cells forming this type of lesion showed diffuse granular staining for NOD2 in the cytoplasm, with inconsistent punctate NOD1 + immunolabeling. NOD1 and NOD2 detect conserved motifs of the bacterial cell wall present in the cell cytosol (41), which are released as a consequence of PGN remodeling during bacterial division or digestion by host enzymes (42). Thus, the expression of NOD1 and NOD2 by the macrophages forming the multibacillary lesions indicates the presence of free cell-wall fragments in the cell cytosol. Since MAP prevents lysosomal degradation and, most probably, replicates within the phagosome (43, 44), these components may be released during the multiplication of the mycobacteria (45, 46) and actively secreted into the cytosol for NOD-dependent detection (47, 48). Although the precise mechanism by which NOD1 and NOD2 interact with their respective ligands is not completely understood (49), the punctate to granular staining observed for both markers inside the macrophages could indicate a relocation of these cytosolic receptors to the MAP-containing endosomes (50, 51). The scarce immunoreactivity observed for NOD1 suggests a marginal response of this receptor even to high concentrations of MAP, as previously reported (27). However, caution should be taken when interpreting the results obtained from *in vitro* studies involving cultured macrophages, as they may not fully represent the complexity of the innate immune response that takes place within a well-developed granuloma. Beyond PGN recognition, NOD1 and NOD2 participate in the immunological response against pathogenic bacteria in other ways, including the regulation of adaptive immunity (38, 49). In fact, evidence suggests that NOD1 and NOD2 collaborate with TLRs in shaping the immunological response during mycobacterial infection (52, 53). Recent studies have identified a high expression of TLR4 in cattle with multibacillary lesions (14, 17). In this sense, excessive TLR4 signaling may lead to a downregulation of IL-12 expression via NOD2 to prevent excessive inflammation (14, 17, 54). Therefore, the NOD-mediated response in this pathological form may not be protective but rather contribute to the polarization of the local immunity toward an ineffective anti-inflammatory response (55).

Focal lesions have been observed both in the early stages of infection and in adult cattle with subclinical infection, leading to the hypothesis that they represent persistent latent forms (6). On the other hand, diffuse paucibacillary lesions are infrequent pathological forms observed in animals with clinical disease, but their pathogenesis remains largely elusive (37, 40). Contrary to multibacillary forms, focal and diffuse paucibacillary lesions are marked by a robust local pro-inflammatory response (12, 13), with a few AFB detected in the granulomas (6). The macrophages in these lesions do not show immunoreactivity for NOD1 or NOD2, indicating undetectable levels of NLR proteins. This suggests the possibility of a lack of antigenic stimulation in the granulomas (56), likely due to the limited multiplication of MAP within macrophages (45). As observed with other mycobacteria (57), spheroplasts or non-replicative forms of MAP have been identified in cows with paratuberculosis (58, 59), but their role in disease pathogenesis is still unclear. González et al. (6) suggested the presence of these forms in focal and diffuse paucibacillary lesions, where detecting AFB using Ziehl-Neelsen staining or immunohistochemical methods is frequently unsuccessful. Stress conditions within the granuloma, such as the production of nitric oxide (NO) by macrophages (12), could activate a dormant phenotype of MAP (60), similar to what has been reported for *Mycobacterium tuberculosis* (61, 62). This activation may enable the pathogen to persist inside macrophages for extended periods (63). Conversely, several studies have indicated that the activation of NOD1 and NOD2 contributes to the expression of inducible nitric oxide synthase (iNOS) and tumor necrosis factor-alpha (TNF-*α*) by macrophages during mycobacterial infection (53, 64–66). However, Fernández et al. (12) demonstrated strong TNF-α and iNOS immunolabeling in macrophages associated with focal and diffuse paucibacillary lesions, despite a lack of immunoreactivity for NOD1 and NOD2 observed in the present study. Collectively, these findings suggest that TNF-α and NO production in MAP-associated granulomas occur independently of NOD signaling.

The results of this study highlight the critical role of NOD1 and NOD2 receptors in the development of various pathological forms associated with bovine paratuberculosis. The release of PGN fragments during the multiplication of MAP inside infected macrophages, forming diffuse multibacillary lesions, likely stimulates the expression of NOD2, and to a lesser extent, NOD1. However, excessive TLR4 signaling in this pathological form may favor a Th2 polarization of local immunity via NOD2, creating a microenvironment conducive to bacterial growth, as observed in other studies (12, 14). On the other hand, the presence of non-replicative forms of MAP in focal and diffuse paucibacillary lesions, due to a robust local pro-inflammatory response, could explain the lack of NLR expression by macrophages. The differential expression patterns of NOD1 and NOD2 across different pathological forms of bovine paratuberculosis allow us to better understand the importance of these receptors in the host immune response and their potential role in disease pathogenesis. These findings open the door to new strategies for the treatment and management of bovine paratuberculosis, focused on modulating the immune response and its interaction with MAP.

The relatively small sample size used in the present study limits the ability to fully capture the diversity of factors that may influence lesion development. Additionally, the inability to account for the initial conditions of infection, such as the timing, dose, and route of exposure, represents a challenge in interpreting the results. The study focused on a group of adult female Holstein cattle, but intrinsic animal factors such as genetics, immune response variability, and other potential environmental or physiological influences were not comprehensively considered. These factors could play a significant role in shaping the lesion microenvironment and influencing lesion progression. Therefore, further research with a larger and more diverse animal population, as well as a broader consideration of these intrinsic factors, is essential to improve the understanding of how various variables interact in the development of lesions during chronic infections.

## Data Availability

The raw data supporting the conclusions of this article will be made available by the authors, without undue reservation.

## Supplementary material

SUPPLEMENTARY FIGURE S1 Tissue sections of control and infected cattle showing different types of lesions associated with paratuberculosis infection. **(a)** Control. Lack of granulomatous lesions consistent with MAP infection in the lamina propria (LP) and associated Peyer’s patches of the jejunum. Hematoxylin and eosin (H-E). **(b)** Focal lesions. Multiple small and well-demarcated granulomas (arrows) in the jejunal Peyer’s patches. H-E. Insert: No detectable acid-fast bacilli (AFB) are present in the cytoplasm of the macrophages. Ziehl-Neelsen (Z-N). **(c)** Diffuse paucibacillary lesion. Inflammatory infiltrate in the ileal LP is composed mainly of lymphocytes with small groups of macrophages (arrow) and Langhans giant cells (arrowhead) scattered among them. H-E. Insert: Langhans giant cell contains no detectable AFB. Z-N. **(d)** Diffuse multibacillary lesion. Widespread granulomatous infiltrate (asterisk) composed mainly of macrophages together with some giant cells and low numbers of lymphocytes in the ileal LP. H-E. Insert: Macrophages contain large numbers of AFB. Z-N.

SUPPLEMENTARY FIGURE S2 Immunoblots of NOD1 antibody blocked with TBS-T containing 5% non-fat milk **(a)** and 5% BSA **(b)**. Left reference: Precision Plus Protein™ Kaleidoscope™ Prestained Protein Standards.

## References

[1] Johne H Frothingham L. Ein eigenthumlicher fall von tuberculose beim rind. Deutsche Zeitschriftfur Tiermedizin Vergleichend Pathologie. (1895) 21:438–54.

[2] Rasmussen P Barkema HW Mason S Beaulieu E Hall DC. Economic losses due to Johne’s disease (paratuberculosis) in dairy cattle. J Dairy Sci. (2021) 104:3123–43. doi: 10.3168/JDS.2020-19381, PMID: 33455766

[3] Garcia AB Shalloo L. Invited review: the economic impact and control of paratuberculosis in cattle. J Dairy Sci. (2015) 98:5019–39. doi: 10.3168/JDS.2014-9241, PMID: 26074241

[4] Windsor PA Whittington RJ. Evidence for age susceptibility of cattle to Johne’s disease. Vet J. (2010) 184:37–44. doi: 10.1016/J.TVJL.2009.01.007, PMID: 19246220

[5] Fecteau ME. Paratuberculosis in cattle. Vet Clin North Am Food Anim Pract. (2018) 34:209–22. doi: 10.1016/J.CVFA.2017.10.011, PMID: 29275033

[6] González J Geijo MV García-Pariente C Verna A Corpa JM Reyes LE. Histopathological classification of lesions associated with natural paratuberculosis infection in cattle. J Comp Pathol. (2005) 133:184–96. doi: 10.1016/J.JCPA.2005.04.007 16045917

[7] Vazquez P Garrido JM Juste RA. Specific antibody and interferon-gamma responses associated with Immunopathological forms of bovine Paratuberculosis in slaughtered Friesian cattle. PLoS One. (2013) 8:e64568. doi: 10.1371/JOURNAL.PONE.0064568, PMID: 23724062 PMC3665815

[8] Arsenault RJ Maattanen P Daigle J Potter A Griebel P Napper S. From mouth to macrophage: mechanisms of innate immune subversion by *Mycobacterium avium* subsp. paratuberculosis. Vet Res. (2014) 45:54. doi: 10.1186/1297-9716-45-54, PMID: 24885748 PMC4046017

[9] Kravitz A Pelzer K Sriranganathan N. The Paratuberculosis paradigm examined: A review of host genetic resistance and innate immune fitness in *Mycobacterium avium* subsp. Paratuberculosis infection. Front Vet Sci. (2021) 8:721706. doi: 10.3389/FVETS.2021.721706, PMID: 34485444 PMC8414637

[10] Criado M Benavides J Vallejo R Arteche N Gutiérrez D Ferreras MC. Local assessment of WC1 + γδ T lymphocyte subset in the different types of lesions associated with bovine paratuberculosis. Comp Immunol Microbiol Infect Dis. (2020) 69:101422. doi: 10.1016/J.CIMID.2020.101422, PMID: 31982851

[11] Zapico D Espinosa J Fernández M Criado M Arteche-Villasol N Pérez V. Local assessment of the immunohistochemical expression of Foxp3+ regulatory T lymphocytes in the different pathological forms associated with bovine paratuberculosis. BMC Vet Res. (2022) 18:299. doi: 10.1186/S12917-022-03399-X, PMID: 35927759 PMC9351272

[12] Fernández M Benavides J Castaño P Elguezabal N Fuertes M Royo M. Macrophage subsets within granulomatous intestinal lesions in bovine Paratuberculosis. Vet Pathol. (2017) 54:82–93. doi: 10.1177/0300985816653794, PMID: 27315822

[13] Fernández M Fuertes M Elguezabal N Castaño P Royo M Ferreras MC. Immunohistochemical expression of interferon-γ in different types of granulomatous lesions associated with bovine paratuberculosis. Comp Immunol Microbiol Infect Dis. (2017) 51:1–8. doi: 10.1016/J.CIMID.2017.01.002, PMID: 28504089

[14] Jenvey CJ Shircliff AL Bannantine JP Stabel JR. Phenotypes of macrophages present in the intestine are impacted by stage of disease in cattle naturally infected with *Mycobacterium avium* subsp. paratuberculosis. PLoS One. (2019) 14:e0217649. doi: 10.1371/JOURNAL.PONE.0217649, PMID: 31121006 PMC6532939

[15] Muñoz M Delgado L Verna A Benavides J García-Pariente C Fuertes M. Expression of transforming growth factor-beta 1 (TGF-beta1) in different types of granulomatous lesions in bovine and ovine paratuberculosis. Comp Immunol Microbiol Infect Dis. (2009) 32:239–52. doi: 10.1016/J.CIMID.2007.11.009, PMID: 18242702

[16] Koets A Rutten V Hoek A Van Mil F Müller K Bakker D. Progressive bovine paratuberculosis is associated with local loss of CD4(+) T cells, increased frequency of gamma delta T cells, and related changes in T-cell function. Infect Immun. (2002) 70:3856–64. doi: 10.1128/IAI.70.7.3856-3864.2002, PMID: 12065529 PMC128076

[17] Zapico D Espinosa J Criado M Gutiérrez D del Ferreras MC Benavides J. Immunohistochemical expression of TLR1, TLR2, TLR4, and TLR9 in the different types of lesions associated with bovine paratuberculosis. Vet Pathol. (2024). 62:305–318. doi: 10.1177/03009858241302850, PMID: 39720873

[18] Subharat S Shu D de Lisle GW Buddle BM Wedlock DN. Altered patterns of toll-like receptor gene expression in cull cows infected with *Mycobacterium avium* subsp. paratuberculosis. Vet Immunol Immunopathol. (2012) 145:471–8. doi: 10.1016/J.VETIMM.2011.10.008, PMID: 22078656

[19] Kawai T Akira S. The role of pattern-recognition receptors in innate immunity: update on toll-like receptors. Nat Immunol. (2010) 11:373–84. doi: 10.1038/NI.1863, PMID: 20404851

[20] Takeuchi O Akira S. Pattern recognition receptors and inflammation. Cell. (2010) 140:805–20. doi: 10.1016/J.CELL.2010.01.022 20303872

[21] Almeida-da-Silva CLC Savio LEB Coutinho-Silva R Ojcius DM. The role of NOD-like receptors in innate immunity. Front Immunol. (2023) 14:1122586. doi: 10.3389/FIMMU.2023.1122586/BIBTEX, PMID: 37006312 PMC10050748

[22] Girardin SE Boneca IG Viala J Chamaillard M Labigne A Thomas G. Nod2 is a general sensor of peptidoglycan through muramyl dipeptide (MDP) detection. J Biol Chem. (2003) 278:8869–72. doi: 10.1074/JBC.C200651200, PMID: 12527755

[23] Chamaillard M Hashimoto M Horie Y Masumoto J Qiu S Saab L. An essential role for NOD1 in host recognition of bacterial peptidoglycan containing diaminopimelic acid. Nat Immunol. (2003) 4:702–7. doi: 10.1038/NI945, PMID: 12796777

[24] Maitra A Munshi T Healy J Martin LT Vollmer W Keep NH. Cell wall peptidoglycan in *Mycobacterium tuberculosis*: an Achilles’ heel for the TB-causing pathogen. FEMS Microbiol Rev. (2019) 43:548–75. doi: 10.1093/FEMSRE/FUZ016, PMID: 31183501 PMC6736417

[25] Al Nabhani Z Dietrich G Hugot JP Barreau F. Nod2: the intestinal gate keeper. PLoS Pathog. (2017) 13:e1006177. doi: 10.1371/JOURNAL.PPAT.1006177, PMID: 28253332 PMC5333895

[26] Fernández-García V González-Ramos S Martín-Sanz P del Portillo FG Laparra JM Boscá L. NOD1 in the interplay between microbiota and gastrointestinal immune adaptations. Pharmacol Res. (2021) 171:105775. doi: 10.1016/J.PHRS.2021.105775, PMID: 34273489

[27] Ferwerda G Kullberg BJ de Jong DJ Girardin SE Langenberg DML van Crevel R. *Mycobacterium paratuberculosis* is recognized by toll-like receptors and NOD2. J Leukoc Biol. (2007) 82:1011–8. doi: 10.1189/JLB.0307147, PMID: 17652449

[28] Pott J Basler T Duerr CU Rohde M Goethe R Hornef MW. Internalization-dependent recognition of *Mycobacterium avium* ssp. paratuberculosis by intestinal epithelial cells. Cell Microbiol. (2009) 11:1802–15. doi: 10.1111/J.1462-5822.2009.01372.X, PMID: 19681906

[29] Ariel O Gendron D Dudemaine PL Gévry N Ibeagha-Awemu EM Bissonnette N. Transcriptome profiling of bovine macrophages infected by *Mycobacterium avium* spp. paratuberculosis depicts foam cell and innate immune tolerance phenotypes. Front Immunol. (2020) 10:2874. doi: 10.3389/FIMMU.2019.02874/FULL, PMID: 31969876 PMC6960179

[30] Pinedo PJ Buergelt CD Donovan GA Melendez P Morel L Wu R. Association between CARD15/NOD2 gene polymorphisms and paratuberculosis infection in cattle. Vet Microbiol. (2009) 134:346–52. doi: 10.1016/J.VETMIC.2008.09.052, PMID: 18926647

[31] Küpper JD Brandt HR Erhardt G. Genetic association between NOD2 polymorphism and infection status by *Mycobacterium avium* ssp. paratuberculosis in German Holstein cattle. Anim Genet. (2014) 45:114–6. doi: 10.1111/AGE.12097, PMID: 24320212

[32] Ruiz-Larrañaga O Garrido JM Iriondo M Manzano C Molina E Koets AP. Genetic association between bovine NOD2 polymorphisms and infection by *Mycobacterium avium* subsp. paratuberculosis in Holstein-Friesian cattle. Anim Genet. (2010) 41:652–5. doi: 10.1111/J.1365-2052.2010.02055.X, PMID: 20477790

[33] Nalubamba K Smeed J Gossner A Watkins C Dalziel R Hopkins J. Differential expression of pattern recognition receptors in the three pathological forms of sheep paratuberculosis. Microbes Infect. (2008) 10:598–604. doi: 10.1016/J.MICINF.2008.02.005, PMID: 18457974

[34] Bateman A Martin MJ Orchard S Magrane M Ahmad S Alpi E. UniProt: the universal protein knowledgebase in 2023. Nucleic Acids Res. (2023) 51:D523–31. doi: 10.1093/NAR/GKAC1052, PMID: 36408920 PMC9825514

[35] Gibbons J Chakraborti S. Non parametric statistical inference. 4th ed. Tuscaloosa: Marcel Dekker (2003).

[36] Mishra SK Dubey PK Dhiman A Dubey S Verma D Kaushik AC. Sequence-based structural analysis and evaluation of polymorphism in buffalo nod-like receptor-1 gene. 3 Biotech. (2019) 9:26. doi: 10.1007/S13205-018-1534-2, PMID: 30622864 PMC6314939

[37] Vazquez P Garrido JM Molina E Geijo MV Gomez N Perez V. Latent infections are the most frequent form of paratuberculosis in slaughtered Friesian cattle. Span J Agric Res. (2014) 12:1049–60. doi: 10.5424/SJAR/2014124-5978

[38] Trindade BC Chen GY. NOD1 and NOD2 in inflammatory and infectious diseases. Immunol Rev. (2020) 297:139–61. doi: 10.1111/IMR.12902, PMID: 32677123 PMC8928416

[39] Begg DJ Plain KM de Silva K Gurung R Gunn A Purdie AC. Immunopathological changes and apparent recovery from infection revealed in cattle in an experimental model of Johne’s disease using a lyophilised culture of *Mycobacterium avium* subspecies paratuberculosis. Vet Microbiol. (2018) 219:53–62. doi: 10.1016/J.VETMIC.2018.03.029, PMID: 29778205

[40] Koets A Eda S Sreevatsan S. The within host dynamics of *Mycobacterium avium* ssp. paratuberculosis infection in cattle: where time and place matter. Vet Res. (2015) 46:61. doi: 10.1186/S13567-015-0185-0, PMID: 26092382 PMC4473847

[41] Kaparakis-Liaskos M Goethel A Philpott DJ. NOD1 and NOD2 and the immune response to bacteria. Molec Genet Inflam Bowel Disease. (2019):251–80. doi: 10.1007/978-3-030-28703-0_12

[42] Girardin SE Travassos LH Hervé M Blanot D Boneca IG Philpott DJ. Peptidoglycan molecular requirements allowing detection by Nod1 and Nod2. J Biol Chem. (2003) 278:41702–8. doi: 10.1074/JBC.M307198200, PMID: 12871942

[43] Kuehnel MP Goethe R Habermann A Mueller E Rohde M Griffiths G. Characterization of the intracellular survival of *Mycobacterium avium* ssp. paratuberculosis: phagosomal pH and fusogenicity in J774 macrophages compared with other mycobacteria. Cell Microbiol. (2001) 3:551–66. doi: 10.1046/J.1462-5822.2001.00139.X, PMID: 11488816

[44] Woo SR Heintz JA Albrecht R Barletta RG Czuprynski CJ. Life and death in bovine monocytes: the fate of *Mycobacterium avium* subsp. paratuberculosis. Microb Pathog. (2007) 43:106–13. doi: 10.1016/J.MICPATH.2007.04.004, PMID: 17548182

[45] Boneca IG. The role of peptidoglycan in pathogenesis. Curr Opin Microbiol. (2005) 8:46–53. doi: 10.1016/J.MIB.2004.12.008, PMID: 15694856

[46] Böth D Schneider G Schnell R. Peptidoglycan remodeling in *Mycobacterium tuberculosis*: comparison of structures and catalytic activities of RipA and RipB. J Mol Biol. (2011) 413:247–60. doi: 10.1016/J.JMB.2011.08.014, PMID: 21864539

[47] Sasawatari S Okamura T Kasumi E Tanaka-Furuyama K Yanobu-Takanashi R Shirasawa S. The solute carrier family 15A4 regulates TLR9 and NOD1 functions in the innate immune system and promotes colitis in mice. Gastroenterology. (2011) 140:1513–25. doi: 10.1053/J.GASTRO.2011.01.041, PMID: 21277849

[48] Nakamura N Lill JR Phung Q Jiang Z Bakalarski C De Mazière A. Endosomes are specialized platforms for bacterial sensing and NOD2 signalling. Nature. (2014) 509:240–4. doi: 10.1038/NATURE13133, PMID: 24695226

[49] Caruso R Warner N Inohara N Núñez G. NOD1 and NOD2: signaling, host defense, and inflammatory disease. Immunity. (2014) 41:898–908. doi: 10.1016/J.IMMUNI.2014.12.010, PMID: 25526305 PMC4272446

[50] Barnich N Aguirre JE Reinecker HC Xavier R Podolsky DK. Membrane recruitment of NOD2 in intestinal epithelial cells is essential for nuclear factor-{kappa}B activation in muramyl dipeptide recognition. J Cell Biol. (2005) 170:21–6. doi: 10.1083/JCB.200502153, PMID: 15998797 PMC2171381

[51] Kufer TA Kremmer E Adam AC Philpott DJ Sansonetti PJ. The pattern-recognition molecule Nod1 is localized at the plasma membrane at sites of bacterial interaction. Cell Microbiol. (2008) 10:477–86. doi: 10.1111/J.1462-5822.2007.01062.X, PMID: 17970764

[52] Dubé JY Behr MA. A NOD to the bond between NOD2 and mycobacteria. PLoS Pathog. (2023) 19:e1011389. doi: 10.1371/JOURNAL.PPAT.1011389, PMID: 37262021 PMC10234563

[53] Lee JY Hwang EH Kim DJ Oh SM Lee KB Shin SJ. The role of nucleotide-binding oligomerization domain 1 during cytokine production by macrophages in response to *Mycobacterium tuberculosis* infection. Immunobiology. (2016) 221:70–5. doi: 10.1016/J.IMBIO.2015.07.020, PMID: 26255090

[54] Kim H Zhao Q Zheng H Li X Zhang T Ma X. A novel crosstalk between TLR4- and NOD2-mediated signaling in the regulation of intestinal inflammation. Sci Rep. (2015) 5:1–17. doi: 10.1038/srep12018, PMID: 26153766 PMC4495563

[55] Méndez-Samperio P. Role of interleukin-12 family cytokines in the cellular response to mycobacterial disease. Int J Infect Dis. (2010) 14:e366–71. doi: 10.1016/J.IJID.2009.06.022, PMID: 19762261

[56] Feerick CL McKernan DP. Understanding the regulation of pattern recognition receptors in inflammatory diseases – a ‘nod’ in the right direction. Immunology. (2017) 150:237–47. doi: 10.1111/IMM.12677, PMID: 27706808 PMC5290251

[57] Beran V Havelkova M Kaustova J Dvorska L Pavlik I. Cell wall deficient forms of mycobacteria: A review. Vet Med (Praha). (2006) 51:365–89. doi: 10.17221/5557-VETMED

[58] Hulten K Karttunen TJ El-Zimaity HMT Naser SA Collins MT Graham DY. Identification of cell wall deficient forms of *M. avium* subsp. paratuberculosis in paraffin embedded tissues from animals with Johne’s disease by in situ hybridization. J Microbiol Methods. (2000) 42:185–95. doi: 10.1016/S0167-7012(00)00185-8, PMID: 11018275

[59] Hines ME Styer EL. Preliminary characterization of chemically generated *Mycobacterium avium* subsp. paratuberculosis cell wall deficient forms (Spheroplasts). Vet Microbiol. (2003) 95:247–58. doi: 10.1016/S0378-1135(03)00185-8, PMID: 12935751

[60] Abdissa K Ruangkiattikul N Ahrend W Nerlich A Beineke A Laarmann K. Relevance of inducible nitric oxide synthase for immune control of *Mycobacterium avium* subspecies paratuberculosis infection in mice. Virulence. (2020) 11:465–81. doi: 10.1080/21505594.2020.1763055, PMID: 32408806 PMC7239028

[61] Khabibullina NF Kutuzova DM Burmistrova IA Lyadova IV. The biological and clinical aspects of a latent tuberculosis infection. Trop Med Infect Dis. (2022) 7:48. doi: 10.3390/TROPICALMED7030048, PMID: 35324595 PMC8955876

[62] Voskuil MI Schnappinger D Visconti KC Harrell MI Dolganov GM Sherman DR. Inhibition of respiration by nitric oxide induces a *Mycobacterium tuberculosis* dormancy program. J Exp Med. (2003) 198:705–13. doi: 10.1084/JEM.20030205, PMID: 12953092 PMC2194188

[63] Markova N. Cell Wall deficiency in mycobacteria: latency and persistence. Understanding Tuberculosis. (2012). 193–216. doi: 10.5772/30919

[64] Brooks MN Rajaram MVS Azad AK Amer AO Valdivia-Arenas MA Park JH. NOD2 controls the nature of the inflammatory response and subsequent fate of mycobacterium tuberculosis and *M. bovis* BCG in human macrophages. Cell Microbiol. (2011) 13:402–18. doi: 10.1111/J.1462-5822.2010.01544.X, PMID: 21040358 PMC3259431

[65] Landes MB Rajaram MVS Nguyen H Schlesinger LS. Role for NOD2 in *Mycobacterium tuberculosis*-induced iNOS expression and NO production in human macrophages. J Leukoc Biol. (2015) 97:1111–9. doi: 10.1189/JLB.3A1114-557R, PMID: 25801769 PMC4438743

[66] Lee JY Lee MS Kim DJ Yang SJ Lee SJ Noh EJ. Nucleotide-binding oligomerization domain 2 contributes to limiting growth of *Mycobacterium abscessus* in the lung of mice by regulating cytokines and nitric oxide production. Front Immunol. (2017) 8:1477. doi: 10.3389/FIMMU.2017.01477, PMID: 29163541 PMC5681718

