# Supplementary figures and images for: A nod to paratuberculosis: NOD1 and NOD2 expression in the pathological spectrum of Mycobacterium avium subsp. paratuberculosis infection in cattle
Source: Front Vet Sci. 2025 May 13;12:1549056. doi: 10.3389/fvets.2025.1549056 (PMC12106534; doi:10.3389/fvets.2025.1549056)

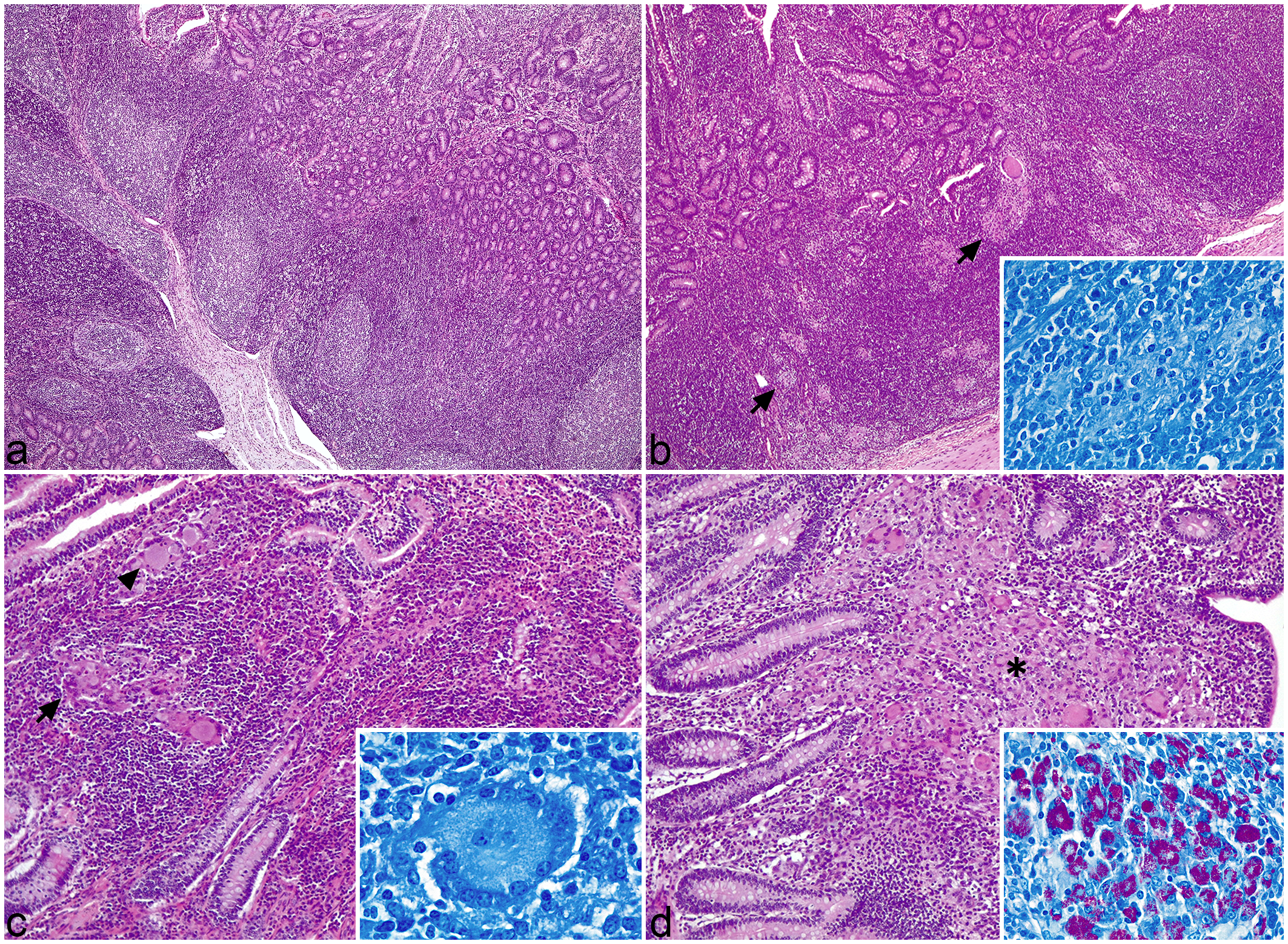

Supplement: SUPPLEMENTARY FIGURE S1 — Tissue sections of control and infected cattle showing different types of lesions associated with paratuberculosis infection. (a) Control. Lack of granulomatous lesions consistent with MAP infection in the lamina propria (LP) and associated Peyer’s patches of the jejunum. Hematoxylin and eosin (H-E). (b) Focal lesions. Multiple small and well-demarcated granulomas (arrows) in the jejunal Peyer’s patches. H-E. Insert: No detectable acid-fast bacilli (AFB) are present in the cytoplasm of the macrophages. Ziehl-Neelsen (Z-N). (c) Diffuse paucibacillary lesion. Inflammatory infiltrate in the ileal LP is composed mainly of lymphocytes with small groups of macrophages (arrow) and Langhans giant cells (arrowhead) scattered among them. H-E. Insert: Langhans giant cell contains no detectable AFB. Z-N. (d) Diffuse multibacillary lesion. Widespread granulomatous infiltrate (asterisk) composed mainly of macrophages together with some giant cells and low numbers of lymphocytes in the ileal LP. H-E. Insert: Macrophages contain large numbers of AFB. Z-N. [file Image_1.tif]

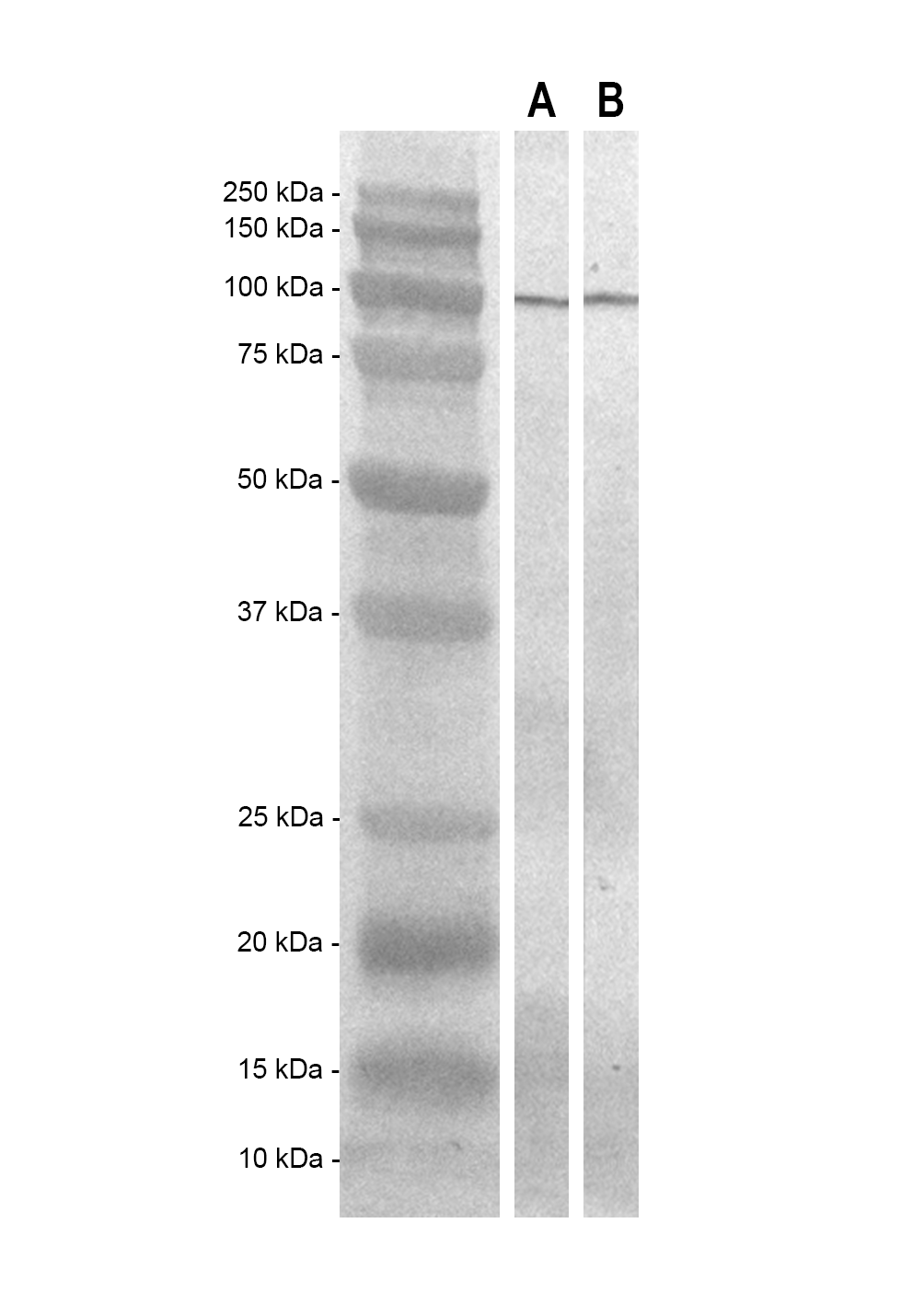

Supplement: SUPPLEMENTARY FIGURE S2 — Immunoblots of NOD1 antibody blocked with TBS-T containing 5% non-fat milk (a) and 5% BSA (b). Left reference: Precision Plus Protein™ Kaleidoscope™ Prestained Protein Standards. [file Image_2.tif]
